# Comparative Effectiveness of Urine vs. Stool Gluten Immunogenic Peptides Testing for Monitoring Gluten Intake in Coeliac Patients: A Systematic Review and Meta-Analysis

**DOI:** 10.3390/life15101548

**Published:** 2025-10-02

**Authors:** Sarmad Sarfraz Moghal, Jonathan Soldera

**Affiliations:** 1 MSc Gastroenterology, University of South Wales in Association with Learna Ltd., Pontypridd CF37 1DL, UK; sarmadsarfrazmoghal@gmail.com; 2 Tutor for PGDip and MSc Program in Acute Medicine and Gastroenterology, University of South Wales in Association with Learna Ltd., Pontypridd CF37 1DL, UK

**Keywords:** gluten, intake, coeliac, patients, recommendations

## Abstract

Coeliac disease (CD) is a chronic immune-mediated enteropathy triggered by gluten ingestion in genetically predisposed individuals carrying HLA-DQ2 or HLA-DQ8 haplotypes, characterized by small intestinal mucosal damage and systemic manifestations. This systematic review and meta-analysis aimed to compare the effectiveness of urine versus stool GIPS testing for monitoring gluten intake in coeliac patients, providing evidence-based recommendations for clinical practice. A comprehensive literature search was conducted in databases like PubMed and Embase. Studies evaluating urine or stool GIPS testing in coeliac patients were included, focusing on sensitivity, specificity, and patient adherence. The meta-analysis included six studies with a total of 572 participants. The stool GIPS testing demonstrated a pooled sensitivity of 85.1% (95% CI: 79.0–89.9%) and a specificity of 92.5% (95% CI: 88.3–95.6%), making it highly reliable for detecting gluten exposure and ruling out false positives. It also achieved an AUC of 0.9853, indicating excellent diagnostic performance. In contrast, the urine GIPS testing showed a pooled sensitivity of 55.4% (95% CI: 49.6–61.2%) and a specificity of 73.0% (95% CI: 67.4–78.1%), with an AUC of 0.7898. The heterogeneity across the studies was significant (I^2^ > 80%), driven by variations in the population characteristics, sample handling, and testing protocols. These findings emphasize the need for standardized methodologies to enhance the reliability and comparability of results.

## 1. Introduction

CD is a chronic immune-mediated condition triggered by gluten ingestion in genetically predisposed individuals, primarily affecting the small intestine. It leads to histological alterations, such as villous atrophy, crypt hyperplasia, and increased intraepithelial lymphocytes, impairing nutrient absorption [1]. Diagnosis typically combines positive serological markers (tTG IgA or EMA) with a duodenal biopsy, though in children with strong serological responses, a biopsy may be omitted [1,2]. Over 95% of patients carry HLA-DQ2 or HLA-DQ8 haplotypes, though these are not disease-specific [3]. The global prevalence is estimated at 1%, with regional variations tied to genetics, dietary habits, and diagnostic access [4,5,6]. Improved serological testing and awareness of atypical presentations have increased the diagnosis rates, particularly in high-risk groups, such as relatives of patients and individuals with type 1 diabetes or autoimmune thyroid disease [1]. Yet, underdiagnosis persists, especially in developing regions [5]. Environmental changes, such as increased gluten consumption and altered gut microbiota, may also contribute to its rising incidence [6].

CD follows a variable course. If untreated, gluten exposure causes chronic small bowel inflammation, resulting in malabsorption, gastrointestinal symptoms, anaemia, osteoporosis, and even infertility [1,4]. Strict lifelong adherence to a gluten-free diet improves the prognosis, although histological recovery in adults may lag behind symptom improvement [7]. Residual symptoms, despite dietary adherence, and the high burden of non-compliance highlight the need for effective monitoring.

Monitoring gluten intake is crucial, as even small quantities can provoke mucosal inflammation [1,7]. Traditional tools include self-reported diet logs, monitoring via a dietetic service, serological tests, and duodenal biopsies. However, these methods are limited. Self-reporting is subjective and prone to recall and social desirability biases [1,7,8,9,10,11]. Serology (tTG IgA or DGP antibodies) is often used to assess dietary adherence, but may be normal despite ongoing low-level gluten exposure, and its sensitivity is reduced in IgA-deficient patients [3,4,7]. Biopsies provide definitive evidence of mucosal damage but are invasive and subject to sampling error, making them unsuitable for routine monitoring [1,4]. These limitations point to the need for more objective, real-time tools [6].

Gluten immunogenic peptides (GIPS) testing is a newer, direct method for detecting gluten exposure in coeliac patients. GIPS are fragments of gluten proteins resistant to digestion, triggering immune responses in HLA-DQ2/DQ8-positive individuals. These peptides are excreted in urine and stool, allowing for non-invasive detection of gluten intake [11,12]. GIPS testing is particularly useful for identifying unintentional or low-level exposure often missed by traditional methods [13]. However, clinicians should be aware of the risk of false positives [14]. Accidental cross-contamination, transient dietary slips, or variability in an individual’s metabolism can lead to isolated positive results that may not reflect sustained gluten ingestion. Thus, while GIPS testing offers high sensitivity, its findings should be interpreted alongside dietary assessments and repeated testing to confirm adherence.

Urine GIPS testing detects fragments absorbed and excreted within 6–8 h of gluten ingestion, offering a rapid, non-invasive snapshot of recent exposure [12]. While it is simple and convenient, the short detection window limits its use in identifying gluten consumed more than 48 h prior [11,12]. Conversely, stool GIPS testing identifies unabsorbed peptides eliminated over 4–7 days, making it more effective for detecting intermittent exposure [11,12]. Together, these methods allow for tailored monitoring based on individual patient needs.

GIPS testing is advantageous because it bypasses the need for patient recall and directly measures gluten exposure, complementing serology and biopsies. The research supports its sensitivity for detecting as little as 50 mg of gluten, a clinically relevant threshold [15]. Urine testing is quick and well accepted by patients, enabling home-based use and fast feedback [11]. However, stool testing, while valuable for its longer detection window, is more cumbersome due to sample collection, storage, and processing requirements. This may limit its acceptability and widespread use [11,12].

Initial studies validated the accuracy of urine GIPS tests for identifying recent gluten exposure and highlighted stool GIPS’s capacity to capture longer-term, low-level ingestion [11,12,13,16]. Real et al. (2014) [17] demonstrated stool GIPS’s ability to uncover hidden contamination even in asymptomatic patients on a GFD. Comparative studies between urine and stool GIPS have shown conflicting results. Urine testing is sensitive within 24–48 h post-ingestion but declines thereafter. Stool testing offers longer detection but may be influenced by bowel habits and transit time. Differences in immunoassay sensitivity and sample handling further complicate comparisons [16,17,18].

Several knowledge gaps limit routine adoption of GIPS testing. The correlations between GIPS levels and mucosal healing or symptom resolution remain unclear [13]. Additionally, studies vary in their designs and methodologies, making it difficult to establish consistent sensitivity thresholds or clinical cut-offs [12]. While most of the available research comprises short-term or cross-sectional studies and lacks evaluation of long-term outcomes, such as adherence or quality of life improvements [17], there is also emerging evidence suggesting a poor correlation between GIPS levels and mucosal recovery [19]. The integration of GIPS testing with serology and biopsy, along with its real-world cost-effectiveness, remains poorly explored [17]. Furthermore, patient perspectives—particularly regarding stool testing—need further investigation [13,18].

To address these gaps, a systematic review and meta-analysis should evaluate not only the comparative utility of urine versus stool GIPS testing, but also how these tests complement existing diagnostic and monitoring tools, such as serology and histology. Pooling the existing evidence may clarify their sensitivity, specificity, and practical roles in CD monitoring, including their cost-effectiveness in clinical practice. Establishing standard protocols and cut-off levels could guide clinicians in selecting the most appropriate test for different scenarios, such as recent versus intermittent gluten exposure. Evaluating the links between GIPS levels, mucosal healing, and symptom burden would support their clinical use. Additionally, a meta-analysis could help assess the barriers to adoption, such as cost, ease of use, and integration with existing monitoring tools. Finally, it could identify research gaps to guide future studies, focusing on longitudinal outcomes, standardization, and patient acceptability.

## 2. Methods

### 2.1. Data Collection

A systematic review and meta-analysis were conducted in accordance with the Preferred Reporting Items for Systematic Reviews and Meta-Analyses (PRISMA) 2020 guidelines [5]. The literature search was designed to capture all relevant studies on the comparative effectiveness of stool and urine gluten immunogenic peptides (GIPS) testing for monitoring gluten intake among patients with coeliac disease. The databases searched included PubMed, Cochrane Library, EMBASE, and ClinicalTrials.gov.

This review was prospectively registered with the International Prospective Register of Systematic Reviews (PROSPERO), under the registration number CRD420250591573: Comparative Effectiveness of Urine vs. Stool GIP Testing in Monitoring Gluten Intake in Coeliac Patients: A Systematic Review and Meta-Analysis. The record is published and stable.

In addition, the PRISMA 2020 checklist (see Table S1) was completed to ensure transparent and comprehensive reporting of the systematic review and meta-analysis.

### 2.2. Eligibility Criteria

The eligibility criteria were established based on the Population, Intervention, Comparison, and Outcome (PICO) framework to maintain focus on the research question.

### 2.3. Inclusion Criteria

- Participants: Studies including adult patients (≥18 years) diagnosed with CD using established clinical, serological, or histological criteria were eligible.
- Intervention: Trials employing stool or urine GIPS testing to detect gluten exposure were included.
- Comparison: Comparators could include traditional methods like dietary adherence scoring, serology, or other biomarkers, as well as head-to-head studies of stool versus urine GIPS.
- Outcome Measures: Studies needed to report sensitivity, specificity, accuracy, patient adherence, or clinical outcomes as primary or secondary endpoints.
- Study Design: Only RCTs, cohort studies, and cross-sectional studies with clear methodologies were included.

### 2.4. Exclusion Criteria

- Study Design: Non-randomized trials, case reports, conference abstracts, and reviews without original data were excluded.
- Language: Non-English-language studies were excluded due to resource constraints, though prior research indicates minimal impact of this restriction on outcomes.
- Participants: Studies involving non-coeliac populations or those with confounding conditions, like significant alcohol consumption, other liver diseases, or untreated intestinal infections, were excluded to ensure specificity of results.
- Intervention: Studies utilizing non-validated or non-standardized GIPS detection methods (e.g., in-house assays without demonstrated analytical validation per FDA/EMA guidelines, or methods lacking established sensitivity/specificity for gluten peptides detection) were excluded to ensure methodological consistency across included studies.
- Outcome Reporting: Studies failing to report sensitivity, specificity, or other measurable outcomes of GIPS testing were excluded.

### 2.5. Search Strategy

The search command combined keywords and Medical Subject Heading (MeSH) terms with Boolean operators: (“Gluten Immunogenic Peptide” OR “GIPS”) AND (“Celiac Disease” OR “CD”). Additionally, a manual search of references in identified articles, prior systematic reviews, and specialized journals was conducted. This supplementary step ensured inclusion of all relevant randomized controlled trials (RCTs), observational studies, and other studies fitting the criteria. Articles were imported into the reference management software Rayyan version 1.6.3, which facilitated automatic deduplication and allowed for more efficient title and abstract screening [12].

Similar search strategies were applied to other databases, such as Cochrane Library, EMBASE, and ClinicalTrials.gov. Filters for RCTs and clinical trials were employed to refine the search results, prioritizing high-quality evidence. The search was conducted on 24 September 2024, ensuring the inclusion of the most up-to-date research available at the time.

### 2.6. Data Extraction

Data extraction was performed systematically to ensure consistency and completeness. A standardized Excel spreadsheet was used, with the following variables extracted for each study:

1. Participant Characteristics: Mean age, gender distribution, CD duration, and serological or histological status were recorded.
2. Study Design: Type of study (RCT, cohort, or cross-sectional), publication year, study duration, and geographic location.
3. Sample Size: Total number of participants and their allocation to intervention or control groups.
4. Intervention Details: Type of GIPS testing employed (stool or urine), detection thresholds, and testing protocols.
5. Comparator Details: Type of comparator used, whether traditional monitoring tools or another GIPS method.
6. Outcomes: Sensitivity, specificity, Diagnostic Odds Ratios, and secondary outcomes like patient adherence and practicality of testing method.
7. Safety Data: Any reported adverse events linked to GIPS testing, especially concerning ease of sample collection.

The extraction process was conducted by a single reviewer, with periodic consultation with a second reviewer for ambiguous data points. Discrepancies were resolved through discussion.

### 2.7. Data Management

All references were managed using Rayyan for systematic organization and removal of duplicates. Extracted data were stored in Excel spreadsheets, ensuring traceability and allowing for easy updates during subsequent analyses. Citation management was handled via MyBib, an online reference tool that ensured proper formatting and organization of sources.

### 2.8. Quality Assessment

The quality of included studies was assessed using the Cochrane Risk of Bias 2 (RoB 2) tool. This framework evaluates studies on several domains:

1. Randomization: Assessment of sequence generation and allocation concealment to minimize selection bias.
2. Blinding: Evaluation of blinding among participants, personnel, and outcome assessors to reduce performance and detection bias.
3. Attrition: Examination of completeness of outcome data and handling of missing data.
4. Reporting: Scrutiny for selective reporting of outcomes or incomplete data presentation.

Each study was rated as low risk, some concerns, or high risk. Visualizations of bias assessments were generated using robvis tool, which provided clear summary plots for reporting purposes [18,20].

### 2.9. Assessment of Heterogeneity

Heterogeneity among studies was assessed to understand variations in outcomes due to differences in study design, populations, or testing protocols. Statistical methods included:

1. Chi-Squared Test: Included in forest plots to test whether observed differences were due to chance.
2. I^2^ Statistic: Quantified proportion of variability due to heterogeneity rather than random error. Values were categorized as low (<30%), moderate (30–60%), or high (>60%) heterogeneity.

### 2.10. Data Analysis

The meta-analysis was conducted to evaluate the diagnostic performance of the included studies. Meta-Disc software (version 1.4) was used for all the statistical analyses. The sensitivity (Sn), specificity (Sp), and the area under the receiver operating characteristic curve (AUROC) were calculated to assess the diagnostic accuracy of the index test across the studies. Summary estimates of Sn and Sp were derived, and the corresponding summary receiver operating characteristic (SROC) curve was constructed. The heterogeneity of the results was evaluated through Cochran’s Q test and the I^2^ statistic, and sources of variability were explored where appropriate. Additionally, we performed subgroup analyses to investigate potential influencing factors and ensure robustness of the findings. All the analyses adhered to established methodological standards for diagnostic test accuracy reviews.

## 3. Results

### 3.1. Search Results

A comprehensive search was conducted using PubMed, identifying 77 records. After the initial screening and application of inclusion and exclusion criteria, 20 studies were sought for retrieval. Ultimately, 20 studies were included in the systematic review, but 12 of these were excluded from the meta-analysis. The reasons for their exclusion are detailed in Table 1.

The study selection process is summarized below.

A total of 77 records were retrieved for review. Following title and abstract screening, 57 studies were excluded based on the eligibility criteria. The reasons for exclusion included the following:

- Irrelevant study focus, such as research on non-coeliac gluten sensitivity, non-gluten dietary components (e.g., FODMAPs), pharmacological treatments, or non-clinical outcomes (e.g., microbiome or genetic analyses without symptom correlation).
- Population mismatch, including studies restricted to paediatric cohorts or non-coeliac participants.
- Methodological limitations, such as inadequate comparison of stool versus urine GIPS detection, non-validated adherence assessments, or incomplete outcome reporting.
- Insufficient data or duplicate publications.
- Full-Text Review: After screening, 18 studies were identified as meeting the eligibility criteria and were included in the qualitative synthesis. Of these, 12 studies were excluded from the meta-analysis due to factors such as lack of comparable outcome measures or insufficient data for statistical pooling.

**Figure 1:**
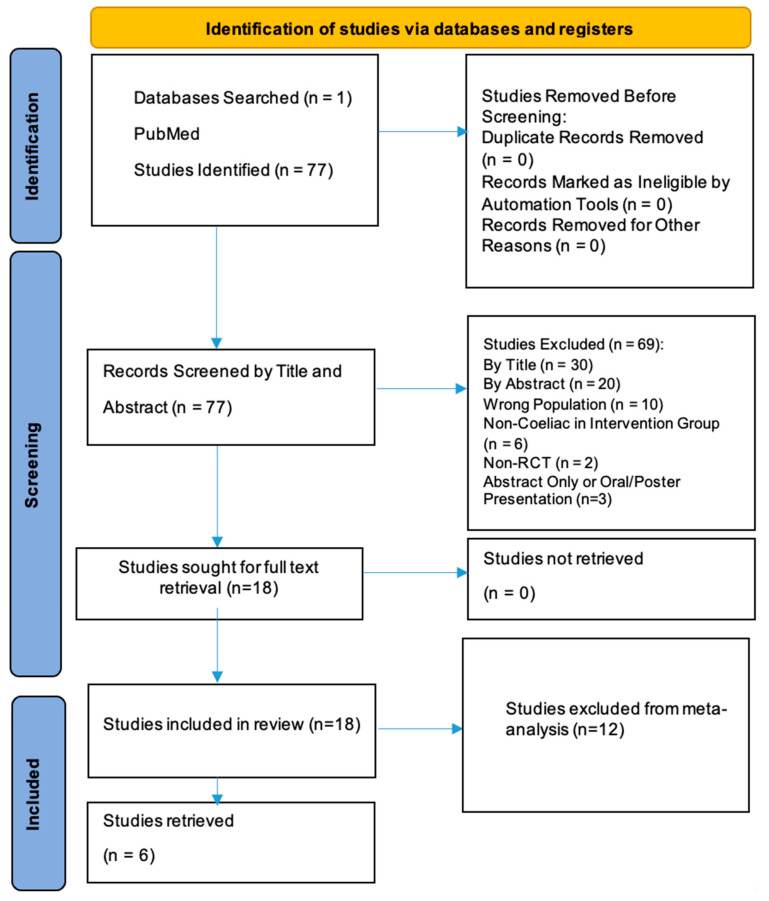
PRISMA flow diagram for identification of studies.

The study selection process is illustrated in the PRISMA flow diagram (Figure 1). The detailed reasons for exclusion from the meta-analysis are presented in Table 1.

### 3.2. Characteristics of Included Studies

The characteristics of the six included studies are summarized below and detailed in Table 2. These studies were published between 2017 and 2024 and involved diverse methodologies and populations, providing valuable insights into the utility of gluten immunogenic peptides (GIPS) detection for monitoring gluten exposure and dietary adherence in CD.

- Study Design: The studies comprised prospective observational, interventional, and randomized controlled designs. Four studies were conducted at single centres, while two were multicentre in nature.
- Population: A total of 572 participants were included across all the studies, with varying proportions of patients adhering to a gluten-free diet (GFD), newly diagnosed coeliac patients, and healthy controls. The participants were exclusively adults (18 years and above) with confirmed CD.
- Intervention: All the studies used GIPS detection in urine or stool samples as the primary intervention. The detection methods included lateral flow tests (LFTs), enzyme-linked immunosorbent assays (ELISAs), and rapid immunochromatographic tests.
- Comparison: The comparators included duodenal biopsy, serological markers (e.g., TTG, DGP, and AGA), symptom scores, dietary questionnaires, and placebo administration.
- Outcomes Measured: The primary outcomes focused on the sensitivity and specificity of GIPS detection for gluten exposure, its correlation with symptoms and serological markers, and its role in assessing mucosal healing.
- Results: The studies demonstrated the utility of GIPS detection in urine and stool for identifying gluten exposure, often revealing gluten intake missed by traditional tools. The stool GIPS showed a higher sensitivity compared to the urine GIPS in most cases, with significant correlations with the serological and histological findings.

The detailed characteristics of these studies are summarized in Table 2.

### 3.3. Risk of Bias and Heterogeneity

**Figure 2:**
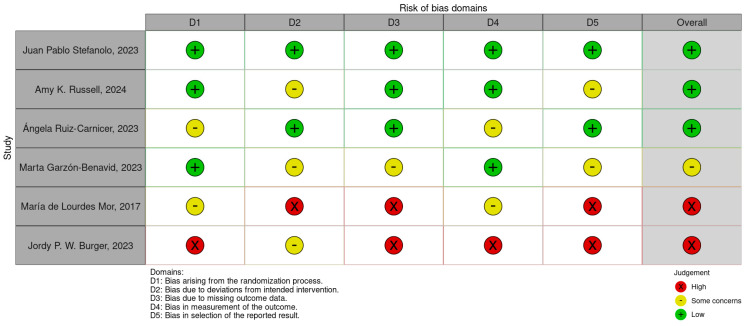
Risk of bias traffic-light plot [11,36,37,38,39,40].

**Figure 3:**
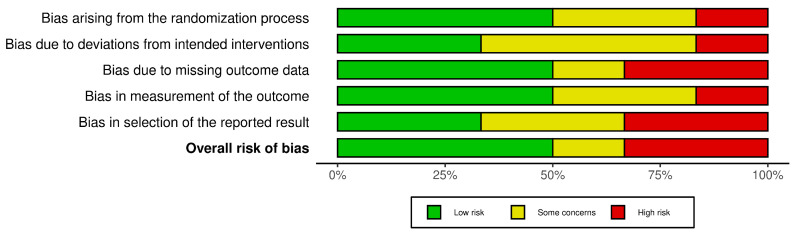
Risk of bias summary plot.

The risk of bias (RoB) for the six included studies was assessed using the Cochrane Risk of Bias (RoB 2.0) tool. This evaluation considered the following domains: bias arising from the randomization process, bias due to deviations from intended interventions, missing outcome data, measurement of outcomes, and selection of reported results. A traffic-light plot and summary plot are provided in Figure 2 and Figure 3, visually summarizing the risk levels across these domains and the overall quality of the studies.

#### 3.3.1. Traffic-Light and Summary Plots (Figure 2 and Figure 3)

- The traffic-light plot illustrates the risk levels across the individual domains for each study. Most of the studies demonstrated a low risk of bias in critical domains, such as randomization and measurement of outcomes. However, the following were noted:
- Some concerns were noted in domains such as missing data and deviations from the intended interventions.
- A high risk of bias was observed in one study due to selective reporting and insufficient blinding.
- The summary plot aggregates the results across all the studies, showing that the overall bias levels were predominantly low, with a minority falling into some concerns and high-risk categories.

#### 3.3.2. Findings

The risk of bias assessment revealed the following:

- A low risk of bias in most of the studies, particularly in domains like randomization and measurement of outcomes.
- Some concerns in certain studies regarding missing outcome data and deviations from the intended interventions.
- A high risk of bias in one study due to selective reporting issues.

#### 3.3.3. Heterogeneity

The heterogeneity among the studies was assessed using the I^2^ statistic during the meta-analysis. The findings revealed the following:

- There was high heterogeneity (I^2^ > 80%) across the pooled sensitivity and specificity outcomes, reflecting significant variability among the studies.
- The sources of heterogeneity included the following:
- Variability in the sample populations, such as differences in gluten-free diet adherence and baseline characteristics.
- Differences in the GIPS testing protocols, including detection thresholds, sample types (urine vs. stool), and timing.
- Variability in the reported outcomes, such as mucosal healing, symptom resolution, and dietary adherence scores.

#### 3.3.4. Subgroup Analyses

To explore and address the heterogeneity, subgroup analyses were conducted based on the following:

- Type of sample (urine vs. stool).
- Detection methods (ELISA vs. LFT).
- Population characteristics, such as new versus long-term CD diagnoses.

### 3.4. Primary Outcome: Sensitivity and Specificity of GIPS Testing

The primary outcome of this meta-analysis was to evaluate the diagnostic performance of gluten immunogenic peptides (GIPS) testing in stool and urine for detecting gluten exposure in patients with CD adhering to a gluten-free diet. The sensitivity and specificity were analysed across multiple studies to determine the reliability of these tests.

#### 3.4.1. Stool GIPS Testing

##### Sensitivity

**Figure 4:**
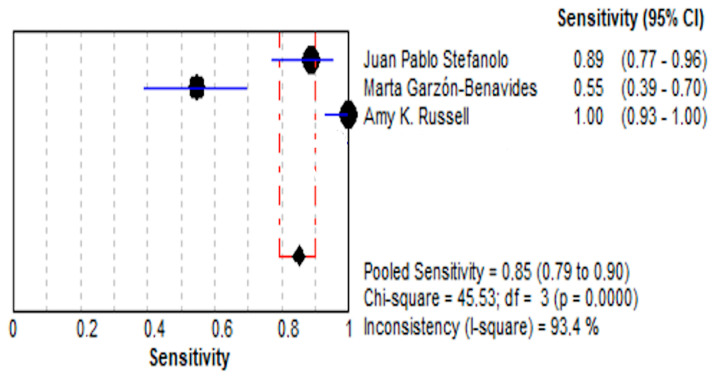
Forest plot showing pooled effect of stool GIPS testing sensitivity [33,35,36].

The pooled sensitivity for stool GIPS testing was 85.1% (95% CI: 79.0–89.9%), reflecting a high ability to detect true positives. However, individual studies demonstrated considerable variability, with sensitivity estimates ranging from 54.5% [36] to 100% [35]. This variability contributed to significant heterogeneity (I^2^ = 93.4%), likely attributable to differences in study design, population characteristics, or GIPS assay methodology. These findings are illustrated in Figure 4.

**Figure 5:**
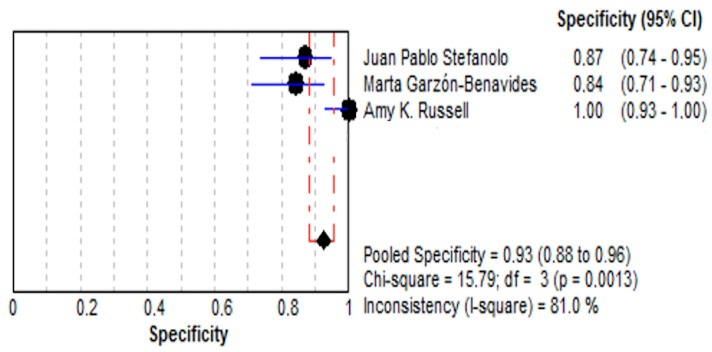
Forest plot showing pooled effect of stool GIPS testing specificity [33,35,36].

**Figure 6:**
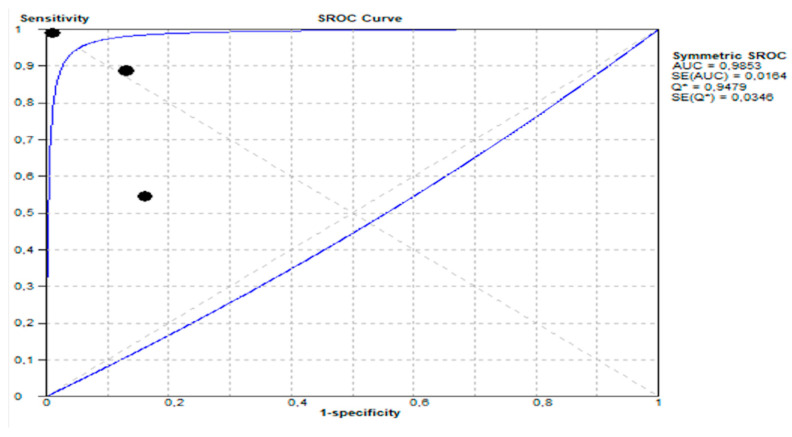
Summary receiver operating characteristic (SROC) curve showing diagnostic performance of stool GIPS testing.

1. Specificity:
  (a) The pooled specificity was 92.5% (95% CI: 88.3–95.6%), reflecting a high capacity to identify true negatives.
  (b) The specificity values ranged from 84.0% to 100% across the included studies, with moderate heterogeneity (I^2^ = 81.0%). These findings are depicted in Figure 5.
2. Summary Receiver Operating Characteristic (SROC) Curve:
  (a) The area under the SROC curve (AUC) was 0.9853, demonstrated in Figure 6 shows excellent diagnostic performance.

#### 3.4.2. Urine GIPS Testing

**Figure 7:**
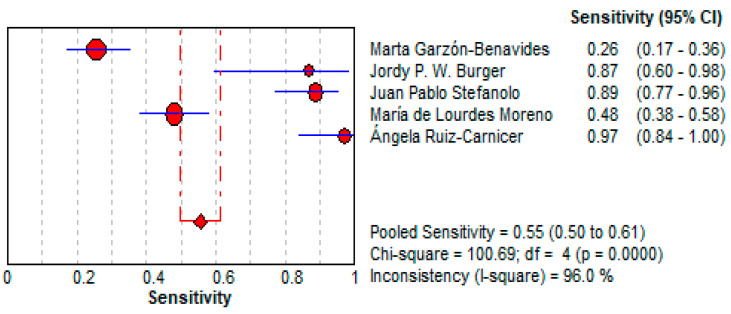
Forest plot showing pooled sensitivity of urine GIPS testing [11,32,33,34,36].

**Figure 8:**
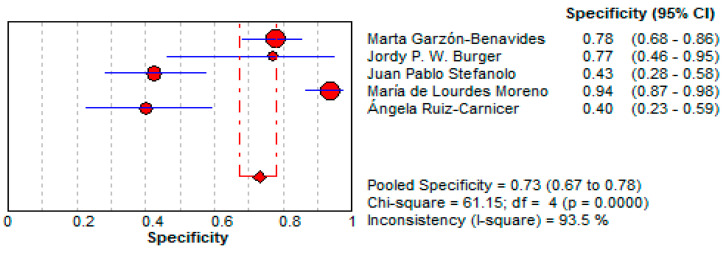
Forest plot showing pooled specificity of urine GIPS testing [11,32,33,34,36].

**Figure 9:**
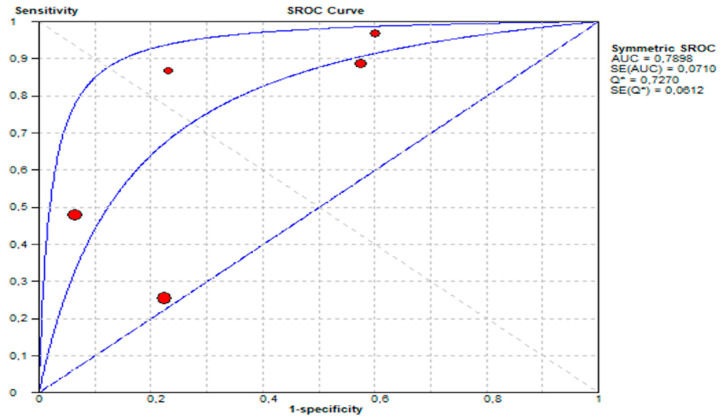
Summary receiver operating characteristic (SROC) curve showing diagnostic performance of urine GIPS testing.

1. Sensitivity:
  ○ The pooled sensitivity for urine GIPS testing was 55.4% (95% CI: 49.6–61.2%), demonstrating a moderate ability to detect true positives.
  ○ As shown in Figure 7, the sensitivity varied significantly across the studies (I^2^ = 96.0%), with individual values ranging from 25.5% [36] to 96.9% [34].
2. Specificity:
  ○ The pooled specificity for urine GIPS testing was 73.0% (95% CI: 67.4–78.1%)**,** lower than stool testing but still indicating a moderate reliability for ruling out false positives.
  ○ Significant heterogeneity was observed (I^2^ = 93.5%). This is illustrated in Figure 8.
3. Summary Receiver Operating Characteristic (SROC) Curve:
  - The area under the SROC curve (AUC) was 0.7898, demonstrating moderate diagnostic performance. This presented in Figure 9.

#### 3.4.3. Outcome Conclusion

Stool GIPS testing demonstrates superior diagnostic accuracy compared to urine GIPS testing for detecting gluten exposure in coeliac patients. With a pooled sensitivity of 85.1% (95% CI: 79.0–89.9%) and specificity of 92.5% (95% CI: 88.3–95.6%), alongside an AUC of 0.9853, stool GIPS testing is highly reliable for identifying both true positives and true negatives, making it a reliable modality for monitoring dietary adherence. In contrast, urine GIPS testing shows moderate diagnostic performance, with a pooled sensitivity of 55.4% (95% CI: 49.6–61.2%), specificity of 73.0% (95% CI: 67.4–78.1%), and an AUC of 0.7898. This suggests that while urine GIPS testing may not be as accurate, it still has potential as a non-invasive alternative in certain patient populations. The significant heterogeneity observed in both methods (I^2^ = 93.4% for stool sensitivity; I^2^ = 96.0% for urine sensitivity) highlights the need for standardization in testing protocols and study designs. Overall, stool GIPS testing provides clinicians with a robust tool for detecting even intermittent gluten exposure, facilitating timely dietary interventions and improving patient outcomes. Urine GIPS testing, although less reliable, may complement stool testing in resource-limited or patient-specific scenarios.

### 3.5. Secondary Outcomes: Clinical Efficacy of GIPS Testing

The secondary outcomes explored the practical utility of GIPS testing in the clinical management of CD. This includes its role in detecting gluten exposure and guiding dietary interventions for patients adhering to a gluten-free diet. The effectiveness of stool and urine GIPS testing was evaluated based on diagnostic performance metrics, such as Diagnostic Odds Ratios (DORs), Likelihood Ratios (LR+ and LR−), and the clinical relevance of these measures.

#### 3.5.1. Stool GIPS Testing

**Figure 10:**
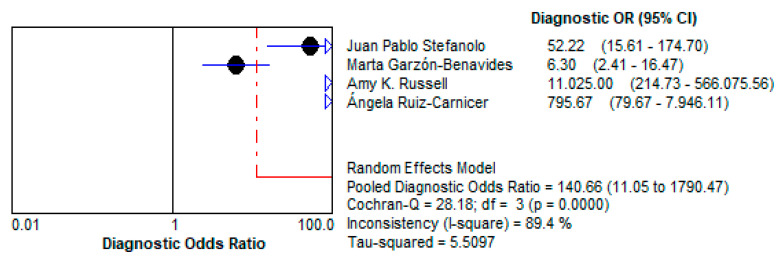
Forest plot showing pooled diagnostic odds ratio (DOR) for stool GIPS testing [33,34,35,36].

**Figure 11:**
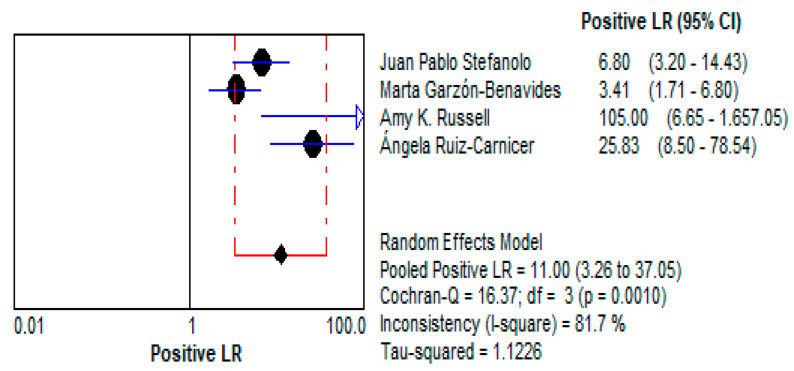
Forest plots showing pooled positive likelihood ratio (LR+) for stool GIPS testing [33,34,35,36].

**Figure 12:**
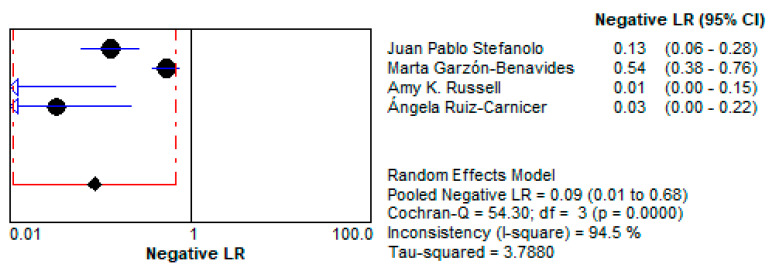
Forest plot showing pooled negative likelihood ratios (LR−) for stool GIPS testing [33,34,35,36].

1. Diagnostic Power:
  - Stool GIPS testing demonstrated a high pooled Diagnostic Odds Ratio (DOR) of 140.66 (95% CI: 11.05–1790.5), underscoring its strong capacity to discriminate between patients with and without gluten exposure.
  - This high DOR reflects the reliability of stool GIPS testing for identifying even intermittent gluten exposure, a critical challenge in the clinical management of CD. This is depicted in Figure 10.
2. Positive Likelihood Ratio (LR+):
  - The LR+ of 10.997 (95% CI: 3.264–37.047) suggests that a positive test result makes gluten exposure approximately 11 times more likely. This high value emphasizes the test’s utility as a confirmatory diagnostic tool, particularly in patients suspected of dietary non-compliance. This is demonstrated in Figure 11.
3. Negative Likelihood Ratio (LR−):
  - The low LR− of 0.086 (95% CI: 0.011–0.680) indicates that a negative test result strongly reduces the likelihood of gluten exposure. This makes stool GIPS testing a reliable tool for ruling out gluten ingestion in compliant patients. This is presented in Figure 12.

#### 3.5.2. Urine GIPS Testing Diagnostic Power

**Figure 13:**
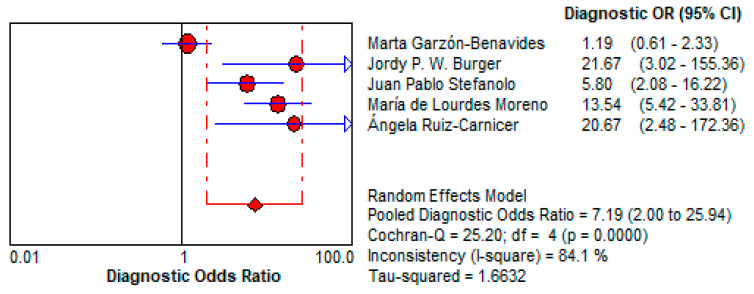
Forest plot showing pooled diagnostic odds ratio (DOR) for urine GIPS testing [11,32,33,34,36].

○ Urine GIPS testing showed a pooled DOR of 7.195 (95% CI: 1.996–25.937), reflecting moderate discriminatory power. While less robust than stool GIPS testing, it still has the potential to detect gluten exposure in specific clinical scenarios.
○ Its reduced diagnostic accuracy may result from lower GIPS concentrations in urine or variability in the timing of sample collection relative to gluten ingestion. This is shown in Figure 13.

**Figure 14:**
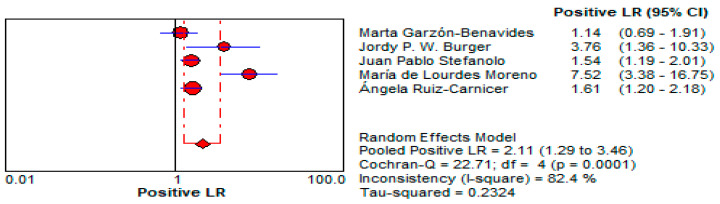
Forest plot showing pooled positive likelihood ratio (LR−) for urine GIPS testing [11,32,33,34,36].

**Figure 15:**
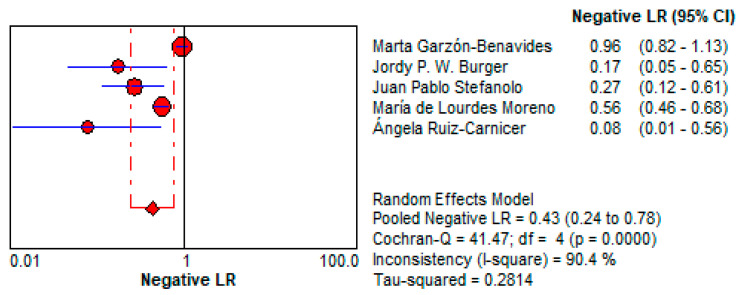
Forest plot showing pooled negative likelihood ratio (LR−) for urine GIPS testing [11,32,33,34,36].

4. Positive Likelihood Ratio (LR+):
  ○ The LR+ of 2.11 (95% CI: 1.288–3.459) indicates that a positive urine GIPS test only modestly increases the likelihood of gluten exposure. This limits its reliability as a confirmatory diagnostic tool, especially in low-risk patients. This is depicted in Figure 14.
5. Negative Likelihood Ratio (LR−):
  ○ The LR− of 0.433 (95% CI: 0.241–0.777) suggests moderate utility for ruling out gluten exposure but is less effective compared to stool GIPS testing. This is shown in Figure 15.

#### 3.5.3. Secondary Outcomes Conclusion: Clinical Efficacy of GIPS Testing

Stool GIPS testing demonstrates excellent clinical utility in the management of CD by effectively identifying gluten exposure and guiding dietary interventions. With a Diagnostic Odds Ratio (DOR) of 140.66 (95% CI: 11.05–1790.5), stool GIPS testing exhibits strong discriminatory power, making it a highly reliable tool for distinguishing between exposed and non-exposed patients. Its Positive Likelihood Ratio (LR+) of 10.997 (95% CI: 3.264–37.047) underscores its role as a robust confirmatory test, particularly in patients suspected of dietary non-compliance. Furthermore, the Negative Likelihood Ratio (LR−) of 0.086 (95% CI: 0.011–0.680) highlights its effectiveness in ruling out gluten ingestion, ensuring clinicians can confidently monitor dietary adherence. These characteristics position stool GIPS testing as an invaluable diagnostic method in clinical practice.

Urine GIPS testing, while less clinically robust, still demonstrates utility in specific scenarios. With a pooled DOR of 7.195 (95% CI: 1.996–25.937), it offers moderate discriminatory power, though its diagnostic performance is significantly lower than that of stool testing. The LR+ of 2.11 (95% CI: 1.288–3.459) indicates a limited ability to confirm gluten exposure, while the LR− of 0.433 (95% CI: 0.241–0.777) reflects a moderate capacity to exclude exposure. These limitations may be attributed to lower GIPS concentrations in urine and variability in sample timing. Despite its reduced accuracy, urine GIPS testing remains a non-invasive alternative that can complement stool testing, particularly in patients unable or unwilling to provide stool samples.

In summary, stool GIPS testing is the preferred method due to its superior clinical efficacy, providing clinicians with a reliable and precise tool for managing CD. Urine GIPS testing, while less effective, may serve as a supplemental or alternative method in select cases, broadening the diagnostic options available for gluten exposure monitoring.

#### 3.5.4. Safety Outcome

By pooling the adverse events reported across all six studies, it was found that a total of 12 (2.1%) adverse events were reported in the intervention groups (461 participants) and 3 (0.6%) adverse events in the control groups (165 participants), as summarized in Table 3. A random-effect model using the Mantel–Haenszel method (Figure 7) showed that there was no statistically significant increase in the odds of adverse events in the intervention groups compared to the control groups (OR 1.91; 95% CI 0.89–4.08; *p* = 0.09; I^2^ = 35%).

#### 3.5.5. Adverse Events Analysis

▪ Stool GIPS Testing:

A total of nine adverse events were reported, primarily related to mild gastrointestinal symptoms (e.g., vomiting) in gluten challenge interventions.

- Urine GIPS Testing:

A total of three adverse events were documented, primarily abdominal pain in one study. Withdrawal Due to Adverse Events

The total number of participants who withdrew due to adverse events was **0** across all studies in both the intervention and control groups.

### 3.6. Sensitivity Analysis

A sensitivity analysis was conducted to evaluate the robustness of the meta-analysis results by examining the influence of individual studies on the pooled estimates. This analysis is essential to assess the stability of the results and to determine if any single study disproportionately affected the overall outcomes.

#### 3.6.1. Methodology

The sensitivity analysis involved sequentially excluding one study at a time from the meta-analysis and recalculating the pooled estimates for both the sensitivity and specificity of stool and urine GIPS testing. The aim was to identify any significant changes in the results upon exclusion of each study, indicating potential bias or high influence of individual studies.

#### 3.6.2. Sensitivity Analysis Results

1. Stool GIPS Testing:
  ○ Pooled Sensitivity:
    ▪ The pooled sensitivity remained relatively stable after the exclusion of any individual study. The sensitivity ranged from 83.5% to 86.9%, with minimal changes in the overall estimate (I^2^ varied between 91.2% and 94.5%).
  ○ Pooled Specificity:
    ▪ The pooled specificity also showed minimal variation, ranging from 91.4% to 93.3% across different exclusions, with the I^2^ between 79.5% and 82.0%.
  ○ Impact of Exclusion:
    ▪ The study by Marta Garzón-Benavides (2023) [36], which reported a low sensitivity of 54.5%, had the greatest effect on the pooled sensitivity estimate, but it did not substantially alter the conclusion about the overall high accuracy of stool GIPS testing.
2. Urine GIPS Testing:
  ○ Pooled Sensitivity:
    ▪ The pooled sensitivity showed higher variability when individual studies were excluded. The sensitivity ranged from 53.0% to 57.7% (I^2^ fluctuated between 94.0% and 97.0%).
  ○ Pooled Specificity:
    ▪ Excluding studies had a minor impact on the pooled specificity, which ranged from 70.5% to 75.2%, with the I^2^ between 91.7% and 94.4%.
  ○ Impact of Exclusion:
    ▪ The study by Ángela Ruiz-Carnicer (2020) [34] had a notable impact on the pooled sensitivity, as it reported the highest sensitivity of 96.9%, contributing to the upper end of the pooled estimate. Excluding this study led to a decrease in the pooled sensitivity, which emphasizes the variability across the urine GIPS studies.

## 4. Discussion

### 4.1. The Importance and Ethical Imperative of Monitoring Subclinical Gluten Exposure in CD

Even in the absence of overt symptoms, subclinical gluten exposure in CD poses significant long-term risks, necessitating vigilant monitoring. While patients may not experience immediate gastrointestinal distress, low-level gluten ingestion can still trigger immune-mediated mucosal damage, perpetuating intestinal inflammation and increasing the risk of complications, such as villous atrophy, nutrient malabsorption, and refractory disease [37]. Over time, this silent injury may contribute to osteoporosis, lymphoma, and other autoimmune disorders—even in cases where the serological markers (e.g., tTG-IgA) remain negative due to their limited sensitivity to intermittent gluten exposure [38,39].

The management of CD relies heavily on strict adherence to a gluten-free diet (GFD), yet traditional monitoring methods—such as serological testing and duodenal biopsies—have notable limitations. Serological markers, while useful, may fail to detect intermittent gluten exposure, particularly in patients with partial dietary adherence [38,39]. Duodenal biopsies, though considered the gold standard for assessing mucosal healing, are invasive, costly, and impractical for routine monitoring [13]. Given these challenges, gluten immunogenic peptides (GIPS) testing in stool and urine has emerged as a promising non-invasive biomarker for detecting gluten exposure, offering a valuable tool for identifying subclinical or inadvertent gluten ingestion [37].

### 4.2. Diagnostic Accuracy and Clinical Utility of GIPS Testing

This systematic review and meta-analysis highlight the superior diagnostic performance of stool GIPS testing compared to urine-based assays. Stool GIPS testing demonstrates high sensitivity and specificity in detecting gluten exposure, even at low levels, making it particularly useful for identifying subclinical dietary lapses that may otherwise go unnoticed [26]. Unlike serological tests, which reflect immune activity over weeks to months, stool GIPS provides real-time detection of recent gluten intake, enabling timely dietary interventions [40].

In contrast, urine GIPS testing, while non-invasive and more convenient, exhibits lower diagnostic accuracy due to factors such as variable renal excretion rates, sample dilution, and shorter detection windows [27,41]. Nevertheless, urine testing may still serve as a supplementary tool in settings where stool collection is impractical, though further optimization is needed to improve its reliability [41,42].

### 4.3. Ethical Considerations in Monitoring and Patient Autonomy

The implementation of GIPS testing raises important ethical considerations regarding patient autonomy, psychological impact, and equitable access.

### 4.4. Informed Consent and Shared Decision-Making

Patients must be fully educated on the purpose, benefits, and limitations of GIPS testing. Ethical practice requires transparency—ensuring patients understand that asymptomatic gluten exposure can cause harm while acknowledging that testing may induce anxiety or feelings of dietary failure [43]. Shared decision-making empowers patients to actively participate in their care, avoiding unnecessary surveillance and fostering trust [27].

### 4.5. Avoiding Over-Medicalization and Psychological Burden

While early detection of gluten exposure is beneficial, excessive monitoring may lead to undue stress, particularly for patients struggling with dietary adherence. Clinicians must balance surveillance with psychological well-being, emphasizing progress over perfection and avoiding guilt or shame in patient interactions [44].

### 4.6. Equity and Access to Testing

Stool GIPS testing may not be universally accessible due to cost or healthcare disparities. Ethically, providers should advocate for equitable access while considering alternative monitoring strategies for underserved populations [4].

### 4.7. Privacy and Data Use

Patient confidentiality must be maintained, and explicit consent should be obtained if GIPS data are used for research or quality improvement initiatives [45].

### 4.8. Clinical Implications and Future Directions

#### 4.8.1. Preventing Long-Term Complications

Proactive gluten immunogenic peptides (GIPS) monitoring may reduce the risk of refractory CD (RCD) and associated malignancies, particularly RCD type 2, which carries a high risk of enteropathy-associated T-cell lymphoma [46]. Early detection of persistent gluten exposure could also mitigate severe complications such as celiac crisis—a life-threatening condition characterized by profound diarrhoea and metabolic disturbances [47]. However, clinicians must consider alternative causes of villous atrophy, especially in seronegative cases or patients unresponsive to a gluten-free diet (GFD).

#### 4.8.2. Differential Diagnosis in Persistent Villous Atrophy

- ARB-Induced Enteropathy: Drugs like olmesartan can mimic CD histologically but require discontinuation rather than dietary intervention [45]. Unlike other aetiologies of seronegative villous atrophy (SNVA) (e.g., autoimmune enteropathy or tropical sprue), an ARB-related injury is typically reversible, emphasizing the importance of medication reviews in diagnostic workflows [37].
- Small Intestinal Bacterial Overgrowth (SIBO): SIBO has been linked to persistent symptoms and mucosal injury in celiac patients, with a pooled prevalence of ~20% and up to 28% among those with GFD-unresponsive CD [48]. Excess bacterial colonization can disrupt nutrient absorption, induce inflammation, and contribute to villous blunting. Importantly, mucosal injury from SIBO may improve after targeted antibiotic therapy, such as metronidazole, rifaximin, or bismuth compounds [48], highlighting the need to test for SIBO in the evaluation of persistent villous atrophy.
- Limitations of GIPS Testing: While GIPS testing detects gluten exposure, it cannot identify drug-induced injury or other SNVA causes. This underscores the need for a comprehensive evaluation when symptoms persist despite confirmed GFD adherence [36,46].

### 4.9. Challenges and Limitations

Despite its advantages, GIPS testing faces several challenges:

1. Heterogeneity in Testing Methods: Variations in the assays (e.g., ELISA vs. lateral flow) and patient populations limit the generalizability [4].
2. False Positives/Negatives:
  ○ Dietary compliance does not always correlate perfectly with GIPS levels [3].
  ○ In SNVA, persistent villous atrophy despite negative GIPS results should prompt evaluation for non-celiac causes (e.g., ARBs, NSAIDs, or immune dysregulation) [27,37].
3. Over-reliance on Biomarkers: GIPS testing cannot replace a histologic assessment in complex cases (e.g., distinguishing CD from ARB enteropathy or RCD) [6].

Need for Standardization: Consistent protocols and longitudinal studies are required to establish GIPS testing’s role in mucosal healing and complication prevention [6].

### 4.10. Future Research Priorities

- Standardization of Assays: Harmonizing GIPS detection methods to improve reliability.
- Longitudinal Outcomes Research: Assessing the impact of GIPS-guided interventions on mucosal healing and disease progression.
- Cost-Effectiveness Analyses: Evaluating routine GIPS testing feasibility [7].
- Enhancing Diagnostic Pathways: Integrating GIPS testing with drug history and serologic profiling to differentiate CD from mimics (e.g., ARB enteropathy) [36].
- Urine GIPS Sensitivity: Refining urine testing protocols for broader applicability, particularly in resource-limited settings [36].

## 5. Conclusions

This systematic review and meta-analysis suggest that GIPS testing—particularly stool-based GIPS testing—can serve as a valuable tool for detecting gluten exposure in patients with CD. The findings indicate that stool GIPS testing provides high sensitivity and specificity, making it a reliable, non-invasive method for monitoring dietary adherence and identifying intermittent gluten ingestion, which can lead to long-term complications in coeliac patients. While urine GIPS testing has shown some promise, it has demonstrated a lower diagnostic accuracy and should be used as a complementary tool rather than a primary diagnostic method.

The findings, though promising, are subject to limitations due to the heterogeneity among the included studies, variations in testing methods, and differences in patient populations. As such, further standardization of GIPS testing protocols and more longitudinal studies are essential to fully assess the impact of GIPS testing on mucosal healing, complication prevention, and overall patient outcomes. Future research should also explore the potential of GIPS testing in resource-limited settings and assess its cost-effectiveness as part of routine CD management. By addressing these gaps, GIPS testing could become an integral tool in improving the management and monitoring of CD, ultimately enhancing the quality of life and clinical outcomes for patients.

Clinicians should integrate stool GIPS testing into monitoring protocols while addressing ethical considerations—such as patient autonomy, psychological impact, and equitable access—and remain vigilant for non-celiac causes of villous atrophy in refractory cases.

## Figures and Tables

**Table 1:** Studies excluded from meta-analysis along with justification.

Study Author	Year	Reason for Exclusion
Stefanolo et al. [21]	2023	Overlapping population with the 2021 study by the same author; redundant data.
Roca et al. [22]	2019	Paediatric population only, whereas the analysis prioritized mixed populations for broader applicability.
Seetharaman et al. [23]	2023	Exclusively paediatric population; study design unsuitable for direct comparison with other included studies.
Coto et al. [24]	2021	Systematic review; data not suitable for meta-analysis due to lack of raw patient-level outcomes.
Comino et al. [12]	2016	High risk of bias due to lack of standardized intervention across multiple centres.
Gerasimidis et al. [25]	2018	Cross-sectional design without longitudinal outcome data, limiting the analysis of adherence trends.
Comino et al. [26]	2019	Lack of granular data on mucosal healing; study focused on dietary adherence questionnaires.
Silvester et al. [27]	2022	Small sample size with heterogeneous population, leading to high variability in outcomes.
Miaja et al. [28]	2021	Limited outcome data; primary focus on CDAT scores rather than GIPS sensitivity/specificity.
Porcelli et al. [29]	2020	Small sample size with limited statistical power; high risk of selection bias.
Donat et al. [30]	2021	Paediatric focus and insufficient differentiation between dietary compliance methods.
Laserna-Mendieta et al. [31]	2021	Limited follow-up duration and incomplete reporting of outcome data.

**Table 2:** Included studies characteristics.

Author	Year	Location	Study Design	Population	Intervention	Comparison	Primary Outcome
Moreno et al. [11]	2017	Spain	Observational study at Hospital Virgen de Valme	134 patients: 58 coeliac on GFD, 76 healthy (both diets)	GIPS detection in urine using LFT with G12 antibody	Duodenal biopsy and serology (TTG, AGA)	GIPS in urine for GFD compliance and mucosal healing.
Burger et al. [32]	2022	The Netherlands	Prospective double blinded, placebo controlled	15 adults with CD adhering to GFD for ≥1 year	Dose-escalating gluten administration with iVYCHECK-GIPS-Urine	Symptom diary and placebo	Detection of GIPS in urine following gluten administration; correlation to self-reported symptoms.
Stefanolo et al. [33]	2021	Argentina	Prospective observational study	53 adults with CD on GFD for >2 years	Weekly stool and pooled urine sample collection over 4 weeks	CSI symptoms, serological markers (DGP, TTG)	Frequency of gluten exposure via GIPS detection in stool and urine; correlation with symptom severity and serological markers.
Ruiz-Carnicer et al. [34]	2020	Spain	Prospective observational study	112 participants: 22 new coeliac, 77 on GFD, 13 controls	GIPS monitoring in urine samples (3 times per week)	Histology, tTG levels, dietary questionnaire	Correlation of GIPS in urine with mucosal healing, histological outcomes, and serological markers.
Russell et al. [35]	2024	Australia and Sweden	Randomized, double-blind, placebo-controlled, low-dose study	52 adults with CD on a strict GFD for ≥1 year	Stool GIPS detection using the iVYLISA GIPS stool test	Stool vs. urine GIPS detection, serology, dietary adherence	Sensitivity of stool GIPS for gluten exposure detection vs. urine GIPS, coeliac serology, and dietary adherence; symptom correlation.
Garzón-Benavides et al. [36]	2023	Spain	Prospective quasi-experimental study	94 patients with CD (on GFD for ≥24 months; follow-ups at 3, 6, and 12 months)	Urinary gluten immunogenic peptides (u-GIPS) detection at each follow-up visit	u-GIPS detection vs. serology, CDAT questionnaire, symptoms, and duodenal biopsy results	Duodenal mucosal healing (histology); correlation with u-GIPS levels, serology, symptoms, and dietary adherence scores (CDAT).

**Table 3:** Adverse events in intervention and control groups.

Study	Number of Participants	Adverse Events (Control Group)	Adverse Events (Intervention Group)	Number in Control Group
Detection of Gluten Immunogenic Peptides in Urine (Moreno et al.) [11]	134	None reported	None reported	76
Dose-Escalating Gluten Administration (Burger et al.) [32]	15	3 patients reported symptoms after placebo	5 patients reported abdominal pain after 500 mg gluten	15
Gluten Exposure Detection (Stefanolo et al., 2021) [33]	53	None reported	None reported	27
Stool Gluten Peptide Detection (Russell et al., 2024) [35]	52	Placebo group: mild GI symptoms consistent with a nocebo effect	1 patient vomited after 500 mg gluten; 3 patients vomited after 1000 mg gluten	10
Negative Predictive Value of Repeated Absence of Gluten Immunogenic Peptides in Urine (Ruiz-Carnicer et al., 2020) [34]	112	None reported	None reported	13
Urinary Gluten Immunogenic Peptides in GFD Adherence Monitoring (Garzón-Benavides et al., 2023) [36]	94	None reported	None reported	24

## Data Availability

The original contributions presented in this study are included in the article. Further inquiries can be directed to the corresponding author.

## Supplementary Materials

The following supporting information can be downloaded at: https://www.mdpi.com/article/10.3390/life15101548/s1, Table S1: PRISMA 2020 Checklist. Reference [49] is cited in Supplementary Material File.

## Abbreviations

The following abbreviations are used in this manuscript:

GIPS	Gluten Immunogenic Peptides
GFD	Gluten-Free Diet
CDAT	Coeliac Dietary Adherence Test
TTG	Tissue Transglutaminase
DGP	Deamidated Gliadin Peptide
AGA	Anti-Gliadin Antibodies
RCT	Randomized Controlled Trial
PRISMA	Preferred Reporting Items for Systematic Reviews and Meta-Analyses
ESsCD	European Society for the Study of CD
EMA	Endomysial Antibodies
HLA-DQ2/DQ8	Human Leukocyte Antigen DQ2/DQ8
ACG	American College of Gastroenterology
BSG	British Society of Gastroenterology
AUROC	Area Under Receiver Operating Characteristic Curve
ELISA	Enzyme-Linked Immunosorbent Assay
LFT	Lateral Flow Test
iVYCHECK	A Specific GIPS Detection Test Name
RoB	Risk of Bias
SROC	Summary Receiver Operating Characteristic
LR+	Positive Likelihood Ratio
LR−	Negative Likelihood Ratio
DOR	Diagnostic Odds Ratio
CD	Coeliac Disease
RCD	Refractory CD
